# Supplementary material for: Do Poisonous Plants in Pastures Communicate Their Toxicity? Meta-Study and Evaluation of Poisoning Cases in Central Europe
Source: Animals (Basel). 2023 Dec 8;13(24):3795. doi: 10.3390/ani13243795 (PMC10740430; doi:10.3390/ani13243795)
Supplement: Supplementary file 1 [file animals-13-03795-s001.zip › animals-2661604-supplementary.pdf]

Table S1: Checklist of poisonous plants in pastures in Central Europe and references

<table>
<tr><th>#</th><th>Plant taxa</th><th>Popular name</th><th>Poison</th><th colspan="2">Cattle</th><th>Sheep</th><th>Goats</th><th colspan="3">Horses</th></tr>
<tr><td>1 (1)[2)]</td><td>Acer campestre (juv. + seeds)</td><td>Field maple</td><td>Hypoglycin A [1]</td><td colspan="2"></td><td></td><td></td><td colspan="3"></td></tr>
<tr><td>2 (1)[3)]</td><td>Acer pseudo-platanus (juv. + seeds)</td><td>Sycamore</td><td>Hypoglycin A [2]</td><td colspan="2">Eaten [3]</td><td></td><td></td><td>Season [4]</td><td>Overgra-zing [5]</td><td>Co-ingestion [1]</td></tr>
<tr><td>3 (2)</td><td>Acer negundo (juv. + seeds)</td><td>Common box elder</td><td>Hypoglycin A [6]</td><td colspan="2"></td><td></td><td></td><td colspan="3">Overgrazing [7]*</td></tr>
<tr><td>4 (3)</td><td>Aconitum napellus</td><td>Friar' cap, garden monkshood</td><td>Aconitine [8], mesaconitine [9]</td><td colspan="2">Overgrazing [10]</td><td>Season [11]</td><td></td><td colspan="3"></td></tr>
<tr><td>5 (2)</td><td>Artemisia vulgaris</td><td>Mugwort</td><td>Thujone [8]</td><td colspan="2"></td><td></td><td></td><td colspan="3"></td></tr>
<tr><td>6 (3)</td><td>Barbarea</td><td>Barbara's herb, winter cress</td><td>Gluconasturtiine [8]</td><td colspan="2"></td><td></td><td></td><td colspan="3"></td></tr>
<tr><td>7 (4)</td><td>Berteroa incana</td><td>Hoary alison</td><td>Unknown [12]</td><td colspan="2"></td><td></td><td></td><td colspan="3"></td></tr>
<tr><td>8 (5)</td><td>Bistorta officinalis</td><td>Adderwort, meadwow bistort</td><td>Oxalic acid, catechins [8]</td><td colspan="2"></td><td></td><td></td><td colspan="3"></td></tr>
<tr><td>9 (1)[4)]</td><td>Caltha palustris</td><td>Marsh marigold</td><td>Protoanemonine [8]</td><td colspan="2">Avoided [13]</td><td></td><td></td><td colspan="3">Unknown [14]</td></tr>
<tr><td>10 (6)</td><td>Cardamine pratensis</td><td>Cuckoo flower, lady's smock</td><td>Glucocochlearine [8]</td><td colspan="2"></td><td></td><td></td><td colspan="3"></td></tr>
<tr><td>11 (4)</td><td>Carum carvi</td><td>Caraway</td><td>d-carvone, d-limonene [8]</td><td colspan="2">Overgrazing [15]</td><td></td><td></td><td colspan="3"></td></tr>
<tr><td>12 (5)</td><td>Chenopodium album</td><td>Fat hen</td><td>Oxalic acid [16]</td><td>Season [17]</td><td>Limited choice [18]</td><td>Limited choice [18]</td><td></td><td colspan="3">Unknown [19]</td></tr>
<tr><td>13 (6)</td><td>Cicuta virosa</td><td>Cowbane</td><td>Cicutoxine, cicutoxinine [8]</td><td>Limited choice [20]</td><td>Unknown [21]</td><td></td><td></td><td colspan="3">Unknown [22]</td></tr>
<tr><td>14 (7)</td><td>Colchicum autumnale</td><td>Autumn crocus</td><td>Colchicine [8]</td><td>Limited choice [23]</td><td>Unknown [24]</td><td>Unknown [25,26]</td><td></td><td colspan="3">Avoided [27]</td></tr>
<tr><td>15 (8)</td><td>Cynoglossum officinale</td><td>Hound's tongue</td><td>Cynoglossine, consolidine [8]</td><td colspan="2">Limited choice [28]</td><td></td><td></td><td colspan="3"></td></tr>
<tr><td>16 (9)</td><td>Echium vulgare</td><td>Bugloss</td><td>Heliosupine [9]</td><td colspan="2">Limited choice [29]</td><td></td><td></td><td colspan="3"></td></tr>
<tr><td>17 (7)</td><td>Equisetum arvense</td><td>Common horsetail</td><td>Silicid acid [8], thiaminase [30]</td><td colspan="2"></td><td></td><td></td><td colspan="3"></td></tr>
</table>

#	Plant taxa	Popular name	Poison	Cattle	Sheep	Goats	Horses
18 (10)	*Equisetum palustre*	Horsetail, marsh horsetail	Silicid acid, palustrine [8], thiaminase [30]	Co-ingestion [15]<br>Season [31]	Avoided [32]		Eaten [32]
19 (8)	*Euphorbia*	Spurge	Resin [8], cyanogenic compounds [33] phorbol esters [9]				
20 (9)	*Glechoma hederacea*	Ground ivy	Etherial oil, glechoma-furan [34]				
21 (11)	*Glyceria*	Sweet grass	Cyanogenic compounds [35]	Overgrazing [36] [15]			
22 (2)	*Heracleum mantegazzianum*	Giant hogweed	8-methoxy-psoralen [9]		Eaten [37,38]		
23 (12)	*Heracleum sphondylium*	Hogweed	8-methoxy-psoralen [9]				Limited choice [39]
24 (3)	*Hippocrepis comosa*	Horseshoe vetch	3-nitro-propionic acid [40]	Unknown [40]	Unknown [40]		
25 (13)	*Hypericum triquetrifolium* Synonym of *H. crispum*	Curled-leaved St. John's wort	Hypericine [8]	Limited choice [41]	Limited choice [41]		Limited choice [41]
26 (14)	*Hypericum perforatum*	St. John's wort	Hypericine [8]		Limited choice [42]		
27 (15)	*Hypochaeris radicata*	Cat's ears	Alkaloids, phenolics, saponins [43]				Overgrazing [44]
28 (16)	*Narthecium ossifragum*	Bog asphodel	Narthecine [9]	Season [45]	Limited choice [46]		
29 (17)	*Oenanthe crocata*[6)]	Hemlock water-dropwort[6)]	Oenantho-toxin [9]	Season [47]			
30 (4)	*Papaver*	Poppy	Rhoeadine, Protopin, Berberine [9]	Avoided [48]			Avoided [48]
31 (18)	*Pastinaca sativa*	Parsnip	Anthotoxin, bergaptene, isopimpinellin, imperatorin [49]				Overgrazing [50]*
32 (5)	*Pedicularis palustre*	Lousewort	Aucubine [8]			Eaten [51]	

#	Plant taxa	Popular name	Poison	Cattle		Sheep	Goats	Horses			
33 (6)	*Persicaria/ Polygonum*	Smartweed/ knotgrass	Oxalic acid [8]	Eaten [51]		Eaten [51]	Eaten [51]	Unknown [51]			
34 (19)	*Phalaris*	Canary grass	Gramine, tryptamine [52]			Season [53]					
35 (20)	*Pteridium aquilinium*	Bracken	Cyanogenic glucosides, ptaquiloside, Thiaminase [9]	Season [54]	Overgrazing [55]	Limited choice [54]	Unknown [56]				
36 (21)	*Ranunculus*	Buttercup	Ranunculin [9]	Limited choice [57] [58]		Eaten [59]	Avoided [51][1]	Limited choice [60]			
37 (7)	*Rhinanthus*	Yellow rattle	Aucubine [8]	Unknown [61]		Avoided [62,63]	Eaten [59]	Avoided [64]			
38 (22)	*Rumex*	Dock	Oxalic acid [8]			Limited choice [42]	Eaten [59]	Unknown [19]			
39 (23)	*Senecio alpinus*[5)]	Alpine ragwort	Senecionine [9]	Limited choice [65]	Co-ingestion [66]			Limited choice [67]			
40 (24)	*Senecio jacobaea*[5)]	Tansy ragwort	Senecionine [9]	Limited choice [68]	Overgrazing [69]	Eaten [70,71]		Season [69] [72]	Overgrazing [69]	Co-ingestion [73]	Unknown [74] [75,76]
41 (25)	*Senecio vulgaris*	Common groundsel	Senecionine [9]	Limited choice [29]							
42 (10)	*Setaria*	Bristle grass	Oxalic acid [77]								
43 (26)	*Solanum nigrum*	Black nightshade	Solanine [8]				Unknown [78]				
44 (11)	*Symphytum*	Comfrey	Symphyto-cynoglossin [8], pyrrolizidine-alkaloids [33]								
45 (8)	*Tanacetum vulgare*	Tansy	Thujone [9]			Eaten [79]					
46 (9)	*Thalictrum*	Meadow rue	Benzyliso-chinoline, cyanogenic compounds [80]	Eaten [51]				Eaten [51]			
47 (12)	*Trifolium*	Clover	cyanogenic compounds [80]								
48 (27)	*Trisetum flavescens*	Yellow oat grass	Dihydroxy-cholecalciferol, vitamin D3 [81]	Limited choice [82]		Unknown [83,84]					

#	Plant taxa	Popular name	Poison	Cattle	Sheep	Goats	Horses
49 (10)	*Trollius europaeus*	Globeflower	Ranunculin [9]			Eaten [51]	
50 (28)	*Urtica dioica*	Nettle	Acetylcholine, hista-mine, serotonine [33]	Unknown [85]		Over-grazing [86]*	
51 (11)	*Veratrum album*	White veratrum	Protoveratrine, Germerine, Cyclopamine [9]			Avoided [51]	
52 (13)	*Vicia*	Vetch	γ-L-glutamyl-β-cyano-L-alanine [87]				

Blank fields (n=135) indicate assumed cases of zero poisoning (no report found). Colored fields indicate circumstances of evidence-based cases of poisoning and zero-poisoning (n=73). Colors symbolize number (n) of animal individuals concerned: dark green: n=0; yellow: n<10; light red: n>10; dark red: n>100

"Eaten": no poisoning when the plant species was eaten; "Avoided": no poisoning when the plant species was avoided; "Season": poisoning was associated with seasonally scarce feed; "Limited choice": poisoning was associated with limited choice of feed plant species; "Overgrazing": poisoning was associated with overgrazing; "Co-ingestion": poisoning was associated with co-ingestion of grass; "Unknown": the circumstances were not indicated.

1)This is related to *R. flammula*. In contrast, goats would eat in small portions *R. repens, R. aconitifolius* and *R. ficaria* without harm [59]. Since Ranunculus had been reduced on the level of the genus in this study, it was not possible to differentiate between the exceptional finding that one species of the genus was eaten and three other species were not.

2)Numbered cases of assumed zero poisoning (n_{total}=13). 1=consecutive number of plant species (1)=case 1 of 13 cases.

3)Grey shadowed: numbered cases of proven poisoning in at least one of the four animal species (n_{total}=28). 2=consecutive number of plant species (1)=case 1 of 28 cases.

4)Numbered cases of proven zero-poisoning (n_{total}=11). 9=consecutive number of plant species (1)=case 1 of 11 cases.

5)The genus *Senecio* has been partly renamed in *Jacobaea* [88]. Accordingly, the two following names changed, too: *Senecio alpinus* to *Jacobaea alpinus* (L.) Moench. as well as *Senecio jacobaea* in *Jacobaea vulgaris* Gaertn. However, in both cases, the synonyms had been used here.

*Personal communication

1. Aboling, S.; Scharmann, F.; Bunzel, D. Equine atypical myopathy: selection of sycamore maple seedlings (Acer pseudoplatanus L.) in pasture horses is driven by seedling maturity and might be associated with phenolic compounds. *Veterinary Record* **2020**, doi:10.1136/vr.105736.
2. Voiton. Equine atypical myopathy: A review. *The Veterinary Journal* **2008**, *178*.
3. Engel, A.M.; El-Khatib, A.H.; Klevenhusen, F.; Weiss, M.; Aboling, S.; Sachse, B.; Schäfer, B.; Weigel, S.; Pieper, R.; Fischer-Tenhagen, C. Detection of Hypoglycin A and MCPrG Metabolites in the Milk and Urine of Pasture Dairy Cows after Intake of Sycamore Seedlings. *Journal of Agricultural and Food Chemistry* **2013**, doi:https://doi.org/10.1021/acs.jafc.3c01248.
4. van Galen, G.; Marcillaud Pitel, C.; Saegerman, C.; Patarin, F., Amory, H.; Baily, J.D.; Cassart, D.; Gerber, V.; Hahn, C.; Harris, P.; Keen, J.A.; et al. European outbreaks of atypical myopathy in grazing equids (2006– 2009): spatiotemporal distribution, history and clinical features. *Equine Vet J* **2012**, *44*, 614–620.
5. Votion, D.M. Atypical myopathy: an update. *In Practice* **2016**, *38*, 241-246.
6. Valberg SJ; BT, S.; AD, H. Seasonal pasture myopathy/atypical myopathy in North America associated with ingestion of hypoglycin A within seeds of the box elder tree. *Equine Vet J* **2013**, *45*, 419–426.
7. Aboling, S. Vergiftung durch Ahornkeimlinge. *Personal communication* **2023**.
8. Gessner, O. *Gift- und Arzneipflanzen von Mitteleuropa*; Carl Winter: Heidelberg, 1974; p. 582.
9. Wink, M. Mode of action and toxicology of plant toxins and poisonous plants. *Mitt. Julius Kühn-Inst* **2009**, *421*, 93-112.
10. Puschner, B.; Booth, M.C.; Tor, E.; Odermatt, A. Delphinium alkaloid toxicosis in cattle from Switzerland. In *Poisonous plants and related toxins*; CABI Publishing Wallingford UK: 2004; pp. 38-43.
11. Amdal, S. Planteforgiftning pä sau i Kvaenangen i begynnelsen av mai 1989 [Plant poisoning in sheep in Kvaenangen at the beginning of May 1989]. *Nork Veterinaertidsskrift* **1991**, *103*, 1031.
12. Kröger, S.e.a. Toxicity after ingestion of hoary alyssum (Berteroa incana) containing hay by horses – a case study. In Proceedings of the Congress Proceedings of the 14th Congress of the European Society of Veterinary and Comparative Nutrition, Switzerland, 2010.
13. Ramsbottom, J. Caroli Linnaei pan suecicus. In Proceedings of the Transactions of the Botanical Society of Edinburgh, 1959; pp. 151-167.
14. Miraglia, N.; Costantini, M.; Polidori, M.; Meineri, G.; Peiretti, P.G. Exploitation of a natural pasture by wild horses: comparison between nutritive characteristics of the land and the nutrient requirements of the herds over a 2-year period. *Animal* **2008**, *2*, 410-418, doi:10.1017/S1751731107001474.
15. Aboling, S.; Rottmann, S.; Wolf, P.; Jahn-Falk, D.; Kamphues1, J. Case Report: Complex Plant Poisoning in Heavily Pregnant Heifers in Germany. *Journal of Veterinary Science and Technology* **2014**, *5*, doi:http://dx.doi.org/10.4172/2157-7579.1000178.
16. Fairley, R.A.; Williams, S.J.; Finnigan, C.E.; Greene, M.A.; Robinson, A.E.; Gill, J.M. The occurrence of hypocalcaemia in mid lactation dairy cattle after the consumption of large amounts of fat-hen (Chenopodium album). *New Zealand Veterinary Journal* **2012**, *60*, 261-262.
17. Pearce, O.D. Drought in the west (Brassica campestis and Chenopodium album poisoning in cattle). *Veterinary Record: Journal of the British Veterinary Association* **1975**, *97*, 60.
18. Herweimjer, C.H.; Houter, L.F.D. Poisoning due to fat hen (Chenopodium album) in sheep. *Netherlands Journal of Veterinary Science* **1971**, *4*, 52-54.

19. Priymenko, N.; Domange, C. Two cases of sudden death in the horse. *Le Nouveau Praticien Vétérinaire-Équine* **2012**, 4-65.
20. Janowski, W.; Lewandowski, L. Water hemlock (Cicuta virosa) poisoning in cattle. *Medycyna weterynaryjna* **1955**, *11*, 166-167.
21. Tatar, A.; Ottowski, F. Mass poisoning of cattle with cowbane (Cicuta virosa L.). *Zycie wetarynaryjne* **1967**, *42*, 137-140.
22. Dijkstra, R.G.; Falkena, R. Cicutoxin poisoning in two ponies (Cicuta virosa, cowbane). *Tijdschrift voor Diergeneeskunde* **1980**, *106*, 1037-1039.
23. Schulz, O.; Hommel, H. Colchicum autumnale poisoning in cattle. *Monatshefte fur Veterinarmedizin* **1975**, *30*, 333-334.
24. Debarnot, A. L'intoxication par le Colchique d'automne. L'Ecole Nationale Veterinaire D'Alfort, Paris, 1968.
25. Panariti, E. Meadow saffron (Colchicum autumnale) intoxication in a nomadic Albanian sheep flock. *Veterinary and Human Toxicology* **1996**, *38*, 227-228.
26. Tribunskii, M.P. Colchicum autumnale poisoning in lambs. **1970**, 71-72 pp.
27. Winter, S.; Penker, M.; Kriechbaum, M. Integrating farmers' knowledge on toxic plants and grassland management: a case study on Colchicum autumnale in Austria. *Biodiversity and Conservation* **2011**, *20*, 1763-1787, doi:10.1007/s10531-011-0060-x.
28. Greatorex, J.C. Some Unusual Cases of Plant Poisoning in Animals. *Veterinary Record* **1966**, *78*, 725-727.
29. Moyano, M.R.; Garcla, A.; Rueda, A.; Molina, A.M.; Mendez, A.; Infante, F. Echium vulgare and Senecio vulgaris Poisoning in Fighting Bulls. *J. Vet. Med. A* **2006**, *53*, 24–25.
30. Hünsche, A.K. Untersuchungen zu möglichen Schadwirkungen einer Kontamination von Grundfutter mit getrocknetem Sumpfschachtelhalm (Equisetum palustre) bei Wiederkäuern und Ponys. 2010.
31. Borg, P.J.V. Ecology of Equisetum palustre in Finland, with special reference to its role as a noxious weed. *Annales Botanici Fennici* **1971**, *8*, 93-141.
32. Lange, G. Sicherung wirtschaftlicher Nutzung von Feuchtgrünlandstandorten unter Berücksichtigung der Sporenpflanze Sumpfschachtelhalm **2012**.
33. Frohne, D.; Jensen, U. *Systematik des Pflanzenreichs unter besonderer Berücksichtigung chemischer Merkmale und pflanzlicher Drogen*, 5. Auflage ed.; Wissenschaftl. Verlagsgesellschaft: Stuttgart, 1998; p. 371.
34. Vanselow, R.; Brendieck-Worm, C. Ground-ivy (Glechoma hederacea) and the cause of toxicosis in horses: in search of evidence. *Zeitschrift für Ganzheitliche Tiermedizin* **2012**, *26*, 88-93, doi:10.1055/s-0032-1315014

https://www.sonntag-verlag.com.

35. Puls, R.; Newschwander, F.P.; Greenway , J.A. Cyanide Poisoning from Glyceria grandis S. Wats. ex Gray (Tall Mannagrass) in a British Columbia. *Canadian Veterinary Journal* **1978**, *19*, 264-265.
36. Guilhon, J. L'enseignement de la botanique et la mort subite au pré. *Bulletin de l'Académie Vétérinaire de France* **1988**, *61*, 267-271.
37. Nielsen, C.; Ravn, H.P.; Nentwig, W.; Wade, M. *The giant hogweed best practice manual*; Hoersholm, 2005; p. 44.
38. Vogt-Andersen, U.; Calov, B. Long-term effects of sheep grazing on giant hogweed (Heracleum mantegazzianum). *Hydrobiologia* **1996**, *340*, 277-284.
39. Ivens, P. Hogweed suspected of causing primary photosensitisation in a horse. *Veterinary Record* **2011**, *169*, 81-82, doi:https://doi.org/10.1136/vr.d4472.
40. Simpson, D.J.; Wainwright, S.J.; Hipkin, C.R. Presence of 3-nitropropionic acid, in widely distributed pasture legumes in Britain. *The Veterinary record* **1999**, *145*, 169-171, doi:10.1136/vr.145.6.169.

41. Lang, E.; Sendil, C. Hypericum crispum, die Ursache einer bei Pflanzenfressern in der Türkei beobachteten Lichtkrankheit. *Tierärztliche Umschau* **1971**, *26*, 73-76.
42. Schrader, A.; Schulz, O.; Völker, H.; Puls, H. Recent plant poisoning in ruminants of northern and eastern Germany. Communication from the practice for the practice. *Berl Munch Tierarztl Wochenschr* **2001**, *114*, 218-221.
43. Senguttuvan, J.; Paulsamy, S.; Karthika, K. Phytochemical analysis and evaluation of leaf and root parts of the medicinal herb, Hypochaeris radicata L. for in vitro antioxidant activities. *Asian Pacific Journal of Tropical Biomedicine* **2014**, *4*, S359-S367, doi:https://doi.org/10.12980/APJTB.4.2014C1030.
44. König, K.D., J.; Echelmeyer, J.; Eikelberg, D.; Schwarz, S.; Baumgärtner, W.; Feige, K.; Aboling, S. Field study on Hypochaeris radicata L. in horse pastures in Germany – Australian stringhalt as a climatic and ecological phenomenon. *Pferdeheilkunde–Equine Medicine* **2021**, *37*, 556–568.
45. Malone, F.E.; Kennedy, S.; Reilly, G.A.C.; Woods, F.M. Bog asphodel (Narthecium ossifragum) poisoning in cattle. *Veterinary Record* **1992**, *131*, 100-103.
46. Ford, E.J.H. A PRELIMINARY INVESTIGATION OF PHOTOSENSITIZATION IN SCOTTISH SHEEP. *J. Comp. Path.* **1964**, *74*, 37-45.
47. Wilson, A.L.; Johnston, W.G.; McCuska, H.B.; Bannatyne, C.C. Hemlock water droplot (Oenanthe crocata) poisoning in cattle. *Veterinary Record* **1958**, *70*, 587-590.
48. McNaughton, I.H.; Harper, J.L. Papaver L. *J. Ecol.* **1964**, *52*, 767-793, doi:10.2307/2257860.
49. Stegelmeier, B.L.; Colegate, S.M.; Knoppel, E.L.; A., R.K.; Collert, M.G. Wild parsnip (Pastinaca sativa)-induced photosensitization. *Toxicon : official journal of the International Society on Toxinology* **2019**, *167*, 60–66, doi:10.1016/j.toxicon.2019.06.007.
50. Aboling, S. Photodermatitis beim Pferd durch Pastinak auf der Weide. *Botanische Untersuchung* **2011**, Personal communication.
51. Kotowski, M.; Kotowska, D.; Biró, M.; Babai, D.; Sharifian, A.; Szentes, S.; Łuczaj, Ł.; Molnár, Z. Change in European Forage and Fodder Plant Indicator Sets over the Past 250 Years. *Rangeland Ecology & Management* **2023**, *88*, 159-173, doi:https://doi.org/10.1016/j.rama.2023.02.010.
52. Ulvund, M.J. Chronic poisoning in a lamb grazing Phalaris arundinacea. *Acta Veterinaria Scandinavica* **1985**, *26*, 286.
53. Humphreys, D.J. *Veterinary toxicology*, 3rd ed.; Ballière Tindall: London, 1988; p. 356.
54. Parker, W.H.; McCrea, C.T. Bracken (Pteris aquilina) poisoning of sheep in the North York Moors. *Vet Record* **1965**, *77*, 861-866.
55. Evans, C.E. Bracken poisoning of farm animals. *Veterinary Record* **1964**, *76*, 365-372.
56. Fenwick, G. Bracken (Pteridium aquilinum)—toxic effects and toxic constituents. *Journal of the Science of Food and Agriculture* **1989**, *46*, 147-173.
57. Gunning, V.O. Suspected buttercup poisoning in a Jersey cow. *British Veterinary Journal* **1949**, *105*, 393.
58. Heggstad, E. A fatal combination: fat cattle in late pregnancy on poor pasture with buttercups. *Norsk Veterinærtidsskrift* **1989**, *101*, 935-936.
59. Dietl, W. Weeds of pastures and meadows in the European Alps. In *Biology and ecology of weeds*; Springer: 1982; pp. 375-385.
60. Piekarz, J. Buttercup poisoning in a horse. *Medycyna Weterynaryjna* **1981**, *37*, 658.
61. Nilsson, S.G. Mowing and grazing in late summer - effects on herbs, especially orchids. *Svensk Botanisk Tidskrift* **2018**, *112*, 171-175.
62. Pilkington, M. Experimental aftermath grazing of urban neutral grassland. Aberystwyth, 2000; pp. 165-166.
63. Hellström, K.; Huhta, A.-P.; Rautio, P.; Tuomi, J.; Oksanen, J.; Laine, K. Use of sheep grazing in the restoration of semi-natural meadows in northern Finland. *Applied Vegetation Science* **2003**, *6*, 45-52, doi:https://doi.org/10.1111/j.1654-109X.2003.tb00563.x.

64. Turaj, S.; County, C.; Family, S.; My, G.R. A surprising weed, yellow rattle. *University of New Hampshire Extension* http://www. *fsa. usda. gov/Internet/FSA_File/stnr_nh_06172011_weed. pdf. Accessed: November* **2010**, *28*, 2012.
65. Braun, U.; Linggi, T.; Pospischil, A. Ultrasonographic findings in three cows with chronic ragwort (Senecio alpinus) poisoning. *Veterinary Record* **1999**, *144*, 122-126, doi:https://doi.org/10.1136/vr.144.5.122.
66. Pohlenz, J.; Luthy, J.; Minder, H.; Bivetti, A. Enzootische Leberzirrhose beim Rind, verursacht durch Pyrrolizidinalkaloide nach Aufnahme von Senecio alpinus (Alpenkreuzkraut). *Schweizer Archiv fur Tierheilkunde* **1980**.
67. Grabner, A. Enzootic liver cirrhosis and the brain-liver syndrome in horses poisoned with Senecio alpinus. *Pferdeheilkunde* **1990**, *6*, 119-124.
68. Vockrodt, H. [Plant poisoning in farm animals (Senecio jacobaea and Inula conyza) and its prevention]. *Monatshefte fur Veterinarmedizin* **1973**, *28*, 59-62.
69. Craig, J.F.; Kerney, W.; Timoney, J.F. Ragwort Poisoning in Cattle and Cirrhosis of the Liver in Horses. *Veterinary Record* **1930**, *10*, 159-174.
70. Susanne Ohlsen, M.G., Peter Wohlsein, Aiko Huckauf, Nicola Lenzewski, Sabine Aboling. Grazing Ecology of Sheep and Its Impact on Vegetation and Animal Health on Pastures Dominated by Common Ragwort (Senecio jacobaea L.)—Part 1: Vegetation. *Animals* **2022**, *22*, 1-16, doi:https://doi.org/10.3390/ani12081000
71. Susanne Ohlsen, M.G., Peter Wohlsein, Aiko Huckauf, Nicola Lenzewski, Sabine Aboling. Grazing Ecology of Sheep and Its Impact on Vegetation and Animal Health on Pastures Dominated by Common Ragwort (Senecio jacobaea L.)—Part 2: Animal Health. *Animals* **2022**, *22*, 13, doi:https://doi.org/10.3390/ani1210128.
72. McLintock, J.; Fell, B.F. A case of acute ragwort poisoning in the horse. *Veterinary Record* **1953**, *65*, 319-320.
73. Giles, C.J. Outbreak of ragwort (Senecio jacobaea) poisoning in horses. *Equine Veterinary Journal* **1983**, *15*, 248-250.
74. Barton, A.; Hewicker-Trautwein, M.; Ohnesorge, B. Leberzirrhose durch Jakobs-Kreuzkraut-Intoxikation. *pferde spiegel* **2010**, *13*, 36-39, doi:10.1055/s-0029-1240895.
75. Passemard, B.; Priymenko, N. Equine poisoning by Senecio in France. *REVUE DE MEDECINE VETERINAIRE* **2007**, *158*, 425-430.
76. Brügmann, M.; Niemann, U.; Wiedenfeld, H.; Geburek, F.; Jünnemann, D.F. Fallbericht aus der Pathologie: Tod eines Norwegers. **2006**.
77. Stewart, J.; Liyou, O.; Wilson, G. Bighead in horses - not an ancient disease. *Australian Equine Veterinarian* **2010**, *29*, 55-62.
78. Gunning, O.V. Poisoning in goats by black nightshade (Solanum nigrum). *British Veterinary Journal* **1949**, *105*, 473-474.
79. Angell, G.N. What! Sheep control tansy (Tanacetum)? *Ore. Fmr.* **1950**, *73*, 861-872.
80. Frohne, D.; Pfänder, H.J. *Giftpflanzen. Ein Handbuch für Apotheker, Ärzte, Toxikologen und Biologen*, 4. Auflage ed.; Kiel, 1999.
81. Wlaschitz, S.; Riedelberger, K.; Gruber, A. Goldhaferinduzierte enzootische Kalzinose bei Pferden in der österreichischen Dachstein-Tauern-Region. *Veterinary Medicine Austria* **2006**, *93*, 253-258.
82. Simon, U. Goldhafer und Rinderkalzinose. *Der Tierzüchter* **1980**, *32*, 292-293.
83. Troxler, J.; Jans, F.; Floch, C. Utilization and conservation of dry marginal areas by grazing sheep and suckler cattle. II. Effect on the vegetation. *Revue Suisse d'Agriculture* **1990**, *22*, 231-238.
84. Fatyga, J. Trends in animal production in consecutive and mixed grazing systems of cattle and sheep in the Sudeten. Banská Bystrica, 1990; pp. 290-293.

85. Ludvíková, V.; Pavlů, V.V.; Gaisler, J.; Hejcman, M.; Pavlů, L. Long term defoliation by cattle grazing with and without trampling differently affects soil penetration resistance and plant species composition in Agrostis capillaris grassland. *Agriculture, Ecosystems & Environment* **2014**, *197*, 204-211.
86. Aboling, S. Gesichtsschwellung bei einer Ziege durch Äsung von Brennessel. *Fallberichts-Sammlung* **2022**, Personal communication.
87. Frncoglu, H.K.; Tate, M.; Ünal, S.; Dogruyol, L.; Özcan, I. A selection strategy for low toxin vetches (Vicia sativa spp.). *Turkish Journal of Agriculture and Forestry* **2007**, *31*, 303-311.
88. Gardens, K.R.B. The International Plant Names Index and World Checklist of Vascular Plants 2023. Available online: (accessed on 20231030).
